# Associations between sleep parameters, non-communicable diseases, HIV status and medications in older, rural South Africans

**DOI:** 10.1038/s41598-018-35584-0

**Published:** 2018-11-23

**Authors:** F. Xavier Gómez-Olivé, Julia K. Rohr, Laura C. Roden, Dale E. Rae, Malcolm von Schantz

**Affiliations:** 1 MRC/Wits Rural Public Health and Health Transitions Research Unit (Agincourt), School of Public Health, Faculty of Health Sciences, University of the Witwatersrand, Johannesburg, South Africa; 2 Harvard Center for Population and Development Studies, Harvard T.H. Chan School of Public Health, Harvard University, Cambridge, Massachusetts USA; 3 INDEPTH Network, Accra, Ghana; 4 Department of Molecular and Cell Biology, Faculty of Science, University of Cape Town, Cape Town, South Africa; 5 Division of Exercise Science and Sports Medicine, Department of Human Biology, Faculty of Health Sciences, University of Cape Town, Cape Town, South Africa; 6 Faculty of Health and Medical Sciences, University of Surrey, Guildford, Surrey, UK

## Abstract

As part of the Health and Aging in Africa: A Longitudinal Study of an INDEPTH Community in South Africa (HAALSI), we investigated sleep habits and their interactions with HIV or non-communicable diseases (NCDs) in 5059 participants (median age: 61, interquartile range: 52—71, 54% females). Self-reported sleep duration was 8.2 ± 1.6h, and bed and rise times were 20:48 ± 1:15 and 05:31 ± 1:05 respectively. Ratings of insufficient sleep were associated with older age, lack of formal education, unemployment, and obesity (p < 0.05). Ratings of restless sleep were associated with being older, female, having more education, being unemployed, and single. Hypertension was associated with shorter self-reported sleep duration, poor sleep quality, restless sleep, and periods of stopping breathing during the night (p < 0.05). HIV positive individuals not on antiretroviral treatment (ART) reported more nocturnal awakenings than those on ART (p = 0.029) and HIV negative individuals (p = 0.024), suggesting a negative net effect of untreated infection, but not of ART, on sleep quality. In this cohort, shorter, poor-quality sleep was associated with hypertension, but average self-reported sleep duration was longer than reported in other regions globally. It remains to be determined whether this is particular to this cohort, South Africa in general, or low- to middle-income countries undergoing transition.

## Introduction

While the global prevalence of non-communicable diseases (NCDs) continues to increase, the burden of NCDs is growing at a faster rate in developing countries. Those in earlier stages of epidemiological transition or where the speed of transition is most rapid are thought to be most vulnerable. South Africa is considered to be a developing upper-middle income country, experiencing a demographic and epidemiologic transition, and currently facing a quadruple burden of disease — the simultaneous occurrence of infectious disease epidemics, high prevalence of NCDs, perinatal and maternal health challenges, and high levels of injury and violence-related deaths.

Historically, infectious diseases have represented the primary burden in South Africa, culminating in the HIV epidemic. The total population prevalence of HIV infection is estimated to be 12.2%, higher in females (14.4%) than in males (9.9%)^1^. The prevalence of NCDs, however, is increasing rapidly. In 2015, national data indicated that NCDs such as heart disease, cancer, diabetes and stroke were responsible for 56% of all deaths occurring that year. In fact, type 2 diabetes mellitus (T2DM) is now the second most common cause of death in South Africa (5.4%), after tuberculosis (7.2%)^2^. Because a major risk factor for cardiovascular diseases (CVD) and T2DM is obesity, it is concerning that one third of South African males and two thirds of South African females are overweight or obese^1^.

Although sleep is affected by and affects both infectious diseases and NCDs, it is a frequently neglected aspect of health and well-being. Sleep has been reported to be affected by HIV infection and some of the anti-retroviral treatments (ART) prescribed to treat it^3–7^. For example, there have been reports of sleep disturbances associated with one of the common ARTs for HIV, efavirenz^5,6^. Although some high-income countries have moved away from it as first-line therapy, efavirenz remains one of the drugs included in the first line treatment for HIV in South Africa^8^.

Insufficient and/or mistimed sleep is also a major risk factor for obesity and NCDs such as CVD and T2DM^9^. Several prospective cohort studies indicate that both short and long sleep are associated with higher mortality rates and increased risk for CVD^10–13^. US National Health Interview Survey data indicate that relative to sleeping 7–8 h per night, self-reported short (≤6 h) and long sleep (>9 h) duration are independently associated with increased obesity, T2DM, hypertension, and CVD^14^. An added complication is the association between obesity and obstructive sleep apnoea (OSA), a known cardiovascular risk factor that is additive to the effects of obesity alone^15^.

Few studies have investigated the sleep habits of South Africans and other Africans, let alone the interactions between sleep and the increasingly common chronic conditions associated with HIV and NCDs. The assumption that changes in sleep patterns coincided with the other lifestyle changes underlying epidemiologic transitions is one reason why sleep in communities that are at different stages of the urbanisation process is of considerable interest. A study in hunter-gatherer communities (two in Africa and one in South America) found actigraphy-derived time-in-bed ranging between 6.9 and 8.5 h and total sleep times between 5.7 and 7.1 h^16^. However, the generalisability of these observations has been questioned, on the basis of the marginal living conditions of these communities^17^. A recent study in a transitioning African society showed that objectively measured total sleep time was not affected by having access to electricity, but rather, the timing of sleep was affected^18^.

Collectively, the sleep data available to date on South Africans are variable, which is unsurprising given the diversity of cultures, socio-economic status, living conditions, education attained and employment rates. A recent report^19^ described self-reported sleep habits of urban South Africans living in Soweto. Long self-reported sleep times (8.75 ± 1.72 h) were documented, and 29% of the participants routinely napped during the day. Females reported longer sleep times than males, and self-reported sleep duration was negatively associated with body mass index (BMI) in females older than 40 years of age, and positively associated with blood pressure, regardless of age. While 6.4% of this urban population were confirmed to be HIV positive, the study did not examine any associations between HIV status, ART, and sleep^19^. More recently, a study in a nationally representative sample of older South Africans (>50 y) in the Global Study on Ageing (SAGE) observed ethnic group differences in self-reported sleep duration^20^. This study reported that short sleep was more prevalent in white Africans than non-white Africans even after accounting for covariates such as age, gender, education, wealth status, residence and chronic disease. The study did not investigate any associations between self-reported sleep duration and HIV or NCDs.

The Agincourt Health and Demographic Surveillance System site was established in 1992 in Agincourt sub-district, one of the most deprived rural areas of South Africa, to better understand the demographic and epidemiologic transitions of this rural population^21^. Older individuals (>50 y, n = 4044) were asked two questions to establish the severity of any nocturnal sleep problems and the extent to which the quality and/or quantity of their sleep affected their daytime function. A third of the population (30.2%) reported having severe/extreme nocturnal sleep problems, and 17.9% indicated that their daytime function was severely/extremely affected by their nocturnal sleep problems. Males who reported feeling unrested or unrefreshed during the day had a 2-fold increased mortality risk^22^.

The Health and Aging in Africa: A Longitudinal Study of an INDEPTH Community in South Africa (HAALSI) study investigates NCDs in a similar ageing segment of the Agincourt cohort^23^. Responses to a modified version of the Pittsburgh Sleep Quality Index (PSQI) questionnaire^24^ were captured during this study. The responses to these questions are presented here, and have allowed us to derive information about sleep quality and duration, and relate these to NCDs, as well as HIV status and medication use. Therefore, the aims of this study were to (i) describe aspects of self-reported sleep duration and quality within this cohort of older, rural, black South Africans, and (ii) explore relationships between self-reported sleep duration, timing and quality and HIV status, ART, and NCDs.

## Results

### Characteristics of the study cohort

**Figure 1:**
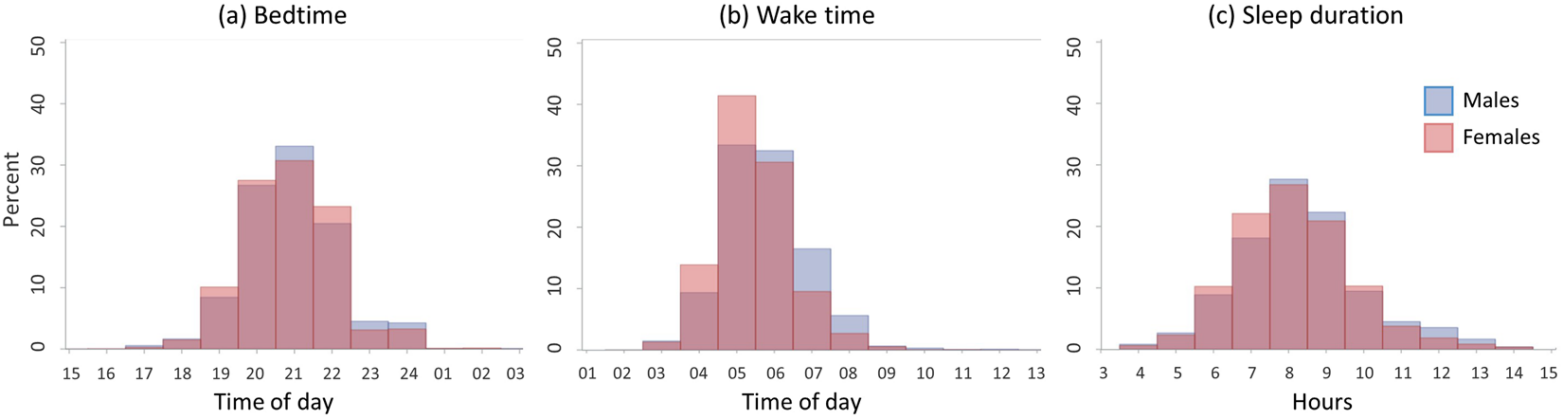
Histograms of self-reported bedtime (**a**), wake time (**b**) and sleep duration (**c**), stratified by sex.

Descriptive data for the 5059 rural participants included in this study are presented in Table 1. Males had on average received more years of education than females (p < 0.001), and more male participants were currently married (p < 0.001), lived in smaller households (p = 0.004) and were employed (p < 0.001). One third of this cohort was classified as obese, 58% hypertensive, 11% diabetic, 23% HIV positive, and almost 15% were taking ART. Females had higher BMIs (p < 0.001) and were more likely to be obese (p < 0.001), hypertensive (p < 0.001) and anaemic (p < 0.001) than the males. Although 94% of this cohort reported having good sleep quality, 32% took longer than 30 minutes to fall asleep at night, 36% felt that their self-reported sleep duration was insufficient, 33% experienced restless sleep, 40% were aware of nocturnal awakenings, 20% reported snoring, 12% reported gasping, and 7% reported that they stop breathing for brief periods in the night. Sleep habits also differed between sexes. Females reported shorter sleep durations (p < 0.001), going to bed earlier (p = 0.023), and waking earlier (p < 0.001) compared to males. Fewer females reported getting sufficient sleep each night (p = 0.005), more females experienced restless sleep (p < 0.001) and nocturnal awakenings (p = 0.008) than males, and more males reported snoring than females (p = 0.008). Figure 1 shows the sex-stratified distributions of bedtime, wake-up time, sleep and total sleep time for all participants.

**Table 1:** Descriptive, demographic, health and sleep characteristics of the cohort.

	All (n = 5059)	Females (n = 2714)	Males (n = 2345)	p-value
Age (y)	61 (52–71)	60 (51–71)	61 (52–71)	0.594
Years of education	4 (1–9)	3 (1–9)	5 (1–9)	<0.001
Married/cohabiting	2575 (50.9%)	973 (35.9%)	1602 (68.3%)	<0.001
Number of people in household	5.3 ± 3.3	5.5 ± 3.3	5.2 ± 3.4	0.004
Employed	805 (16.0%)	362 (13.4%)	443 (19.0%)	<0.001
Household wealth quintile				
1 (poorest)	1046 (20.7%)	544 (20.0%)	502 (21.4%)	0.614
2	1001 (19.8%)	546 (20.1%)	455 (19.4%)	
3	991 (19.6%)	541 (19.9%)	450 (19.2%)	
4	1007 (19.9%)	550 (20.3%)	457 (19.5%)	
5 (wealthiest)	1014 (20.0%)	533 (19.6%)	481 (20.5%)	
BMI (kg·m^−2^)	27.2 ± 6.9	29.3 ± 7.4	24.9 ± 5.4	<0.001
Obese	1384 (29.5%)	1043 (41.2%)	341 (15.8%)	<0.001
Hypertension	2884 (58.4%)	1645 (61.8%)	1239 (54.5%)	<0.001
Dyslipidemia	1861 (43.8%)	1000 (42.9%)	861 (44.9%)	0.203
Diabetes	495 (10.7%)	288 (11.5%)	207 (9.7%)	0.050
Anemia	779 (17.3%)	544 (22.5%)	235 (11.4%)	<0.001
HIV positive	1048 (23.0%)	565 (22.9%)	483 (23.0%)	0.944
HIV+ and on ART	663 (64.0%)	346 (61.8%)	317 (66.6%)	0.108
Sleep duration* (h)	8.23 ± 1.6	8.15 ± 1.6	8.33 ± 1.7	<0.001
Bedtime (h:min)	20:48 ± 1:15	20:46 ± 1:13	20:51 ± 1:18	0.023
Wake time (h:min)	05:31 ± 1:05	05:22 ± 1:00	05:41 ± 1:10	<0.001
Good sleep quality^a^	4724 (93.5%)	2527 (93.2%)	2197 (93.8%)	0.458
Latency > 30 min^b^	1603 (31.8%)	861 (31.9%)	742 (31.8%)	0.952
Sufficient sleep^c^	3241 (64.2%)	1691 (62.4%)	1550 (66.2%)	0.005
Restless sleep	1626 (32.9%)	964 (36.3%)	662 (28.9%)	<0.001
Nocturnal awakenings^c^	2036 (40.3%)	1139 (42.0%)	897 (38.3%)	0.008
Snoring^d^	979 (19.5%)	488 (18.1%)	491 (21.1%)	0.008
Gasping^d^	624 (12.4%)	313 (11.6%)	311 (13.4%)	0.065
Breathing stops^d^	352 (7.0%)	177 (6.6%)	175 (7.5%)	0.203

Data are presented as mean ± standard deviation, median (interquartile range) or count (%). BMI: body mass index; ART: antiretroviral therapy. *Self-reported sleep duration. ^a^“Very good” or “Fairly good”; ^b^Once a week or more; ^c^“Often” or “Very often”; ^d^“Yes”. The p-values represent differences between the two gender groups analyzed using an independent t-test, a Mann-Whitney U test or a Fisher’s Exact test.

### Sleep parameter associations

Table 2 displays associations between demographic and sleep parameters. Self-reported sleep duration was associated with age, sex, employment, marital status, and wealth quintile. Participants aged 50–59 y (p = 0.030) and 60–69 y (p < 0.001) had shorter self-reported sleep durations than 40–49-year-olds; females reported shorter sleep durations than males (p < 0.001); employed individuals and homemakers reported shorter sleep durations than those not working (p < 0.001); married individuals reported shorter sleep durations than those divorced (p = 0.037) or widowed (p = 0.003), and middle, upper-middle and upper income individuals reported shorter sleep durations than individuals in the lowest wealth quintile (p = 0.024, p = 0.001, and p = 0.005, respectively).

**Table 2:** Logistic regression models for associations between demographic and sleep variables.

		Self-reported sleep duration	Bedtime	Wake time	Insufficient sleep	Restless sleep	Snoring
		Quartile 1 - Quartile 4	Quartile 1 - Quartile 4	Quartile 1 - Quartile 4	Sometimes/Rarely/Never sufficient vs. Often/Very often sufficient	Yes vs. No	Yes vs. No
		OR (95% CI)	OR (95% CI)	OR (95% CI)	OR (95% CI)	OR (95% CI)	OR (95% CI)
Age group (y)	50–59 vs 40–49	0.83 (0.70, 0.98)	1.00 (0.85, 1.17)	0.76 (0.64, 0.89)	1.13 (0.93, 1.37)	1.39 (1.12, 1.72)	1.28 (1.00, 1.63)
	60–69 vs 40–49	0.78 (0.65, 0.94)	0.99 (0.83, 1.19)	0.61 (0.51, 0.73)	1.04 (0.84, 1.29)	1.79 (1.42, 2.25)	1.64 (1.26, 2.13)
	70–79 vs 40–49	0.93 (0.76, 1.15)	0.75 (0.61, 0.92)	0.85 (0.69, 1.04)	1.15 (0.91, 1.47)	1.97 (1.53, 2.54)	1.54 (1.14, 2.07)
	80+ vs 40–49	1.24 (0.97, 1.59)	0.53 (0.41, 0.67)	1.18 (0.93, 1.51)	1.37 (1.04, 1.81)	2.46 (1.84, 3.29)	1.80 (1.27, 2.55)
Sex	Female vs Male	0.73 (0.64, 0.83)	0.93 (0.82, 1.05)	0.50 (0.44, 0.57)	1.05 (0.91, 1.21)	1.28 (1.10, 1.49)	0.74 (0.62, 0.88)
BMI	Underweight vs normal	1.01 (0.79, 1.30)	0.71 (0.55, 0.90)	1.06 (0.83, 1.35)	1.09 (0.82, 1.44)	1.27 (0.95, 1.69)	1.34 (0.94, 1.89)
	Overweight vs normal	1.03 (0.90, 1.19)	0.97 (0.84, 1.11)	0.91 (0.79, 1.04)	1.14 (0.97, 1.33)	1.14 (0.97, 1.34)	1.30 (1.07, 1.60)
	Obese vs normal	1.08 (0.93, 1.24)	1.06 (0.92, 1.22)	1.10 (0.95, 1.26)	1.30 (1.11, 1.53)	1.02 (0.86, 1.20)	2.50 (2.05, 3.05)
Education	1–7 years vs No formal education	0.89 (0.78, 1.02)	1.19 (1.05, 1.35)	1.02 (0.9, 1.16)	0.93 (0.81, 1.08)	0.94 (0.81, 1.09)	1.29 (1.08, 1.53)
	8–11 years vs No formal education	0.96 (0.79, 1.16)	1.18 (0.97, 1.43)	1.17 (0.97, 1.42)	0.67 (0.53, 0.85)	0.82 (0.64, 1.04)	1.09 (0.83, 1.44)
	12+ years vs No formal education	0.82 (0.64, 1.03)	1.45 (1.15, 1.83)	0.81 (0.64, 1.02)	0.71 (0.54, 0.94)	0.50 (0.36, 0.70)	1.23 (0.89, 1.70)
Employment	Employed vs Not working	0.57 (0.48, 0.67)	1.00 (0.86, 1.18)	0.43 (0.36, 0.50)	0.94 (0.78, 1.13)	0.76 (0.62, 0.94)	0.99 (0.79, 1.24)
	Homemaker vs Not working	0.45 (0.37, 0.54)	0.88 (0.74, 1.05)	0.96 (0.80, 1.14)	0.34 (0.27, 0.43)	0.86 (0.70, 1.06)	1.20 (0.95, 1.52)
Marital status	Never married vs Currently married	1.18 (0.92, 1.51)	0.95 (0.75, 1.20)	1.57 (1.24, 1.99)	1.10 (0.84, 1.46)	1.14 (0.83, 1.56)	0.82 (0.57, 1.17)
	Separated/divorced vs Currently married	1.20 (1.01, 1.43)	0.85 (0.71, 1.00)	1.35 (1.13, 1.59)	1.15 (0.94, 1.40)	1.38 (1.12, 1.69)	0.76 (0.59, 0.97)
	Widowed vs Currently married	1.23 (1.07, 1.42)	0.79 (0.69, 0.91)	1.20 (1.04, 1.38)	1.13 (0.96, 1.33)	1.33 (1.13, 1.57)	0.66 (0.54, 0.81)
Wealth quintile	2 vs 1 (lowest)	0.94 (0.79, 1.11)	1.21 (1.03, 1.44)	0.92 (0.77, 1.08)	1.11 (0.92, 1.35)	0.91 (0.75, 1.11)	0.86 (0.68, 1.10)
	3 vs 1	0.81 (0.68, 0.97)	1.31 (1.11, 1.56)	0.90 (0.76, 1.07)	0.93 (0.76, 1.14)	1.09 (0.89, 1.33)	0.93 (0.73, 1.18)
	4 vs 1	0.73 (0.61, 0.87)	1.66 (1.40, 1.97)	0.99 (0.83, 1.18)	1.03 (0.84, 1.26)	1.05 (0.85, 1.29)	0.92 (0.72, 1.18)
	5 vs 1	0.76 (0.63, 0.92)	1.83 (1.52, 2.19)	0.99 (0.83, 1.19)	1.05 (0.85, 1.30)	0.89 (0.72, 1.11)	0.88 (0.68, 1.14)

Each of the six sleep parameters (dependent variables) was evaluated in a separate model with the demographic variables serving as independent variables.

OR: odds ratio, CI: confidence interval, BMI: body mass index.

Self-reported bedtime was associated with age, BMI, education level, marital status and wealth quintile. Individuals aged 70–79 y (p = 0.058) and ≥80 y (p < 0.001) had earlier bedtimes than those aged 40–49 y; underweight participants had earlier bedtimes than normal weight participants (p = 0.006); individuals with 1–7 y (p = 0.005) or >12 y (p = 0.002) of education had later bedtimes than those with no formal education; widowed individuals had earlier bedtimes than married individuals (p = 0.001); middle, upper-middle and upper income individuals had later bedtimes than those in the lowest wealth quintile (p = 0.002, p < 0.001 and p < 0.001 respectively). Waking time was associated with age, sex, employment status and marital status. Participants aged 50–69 y woke earlier than 40–49-year-olds (p < 0.001 females woke earlier than males (p < 0.001), employed individuals woke earlier than others (p < 0.001), and currently married individuals woke earlier than those not married (p < 0.001), separated/divorced (p < 0.001), or widowed (p = 0.009).

Ratings of insufficient sleep were associated with age, BMI, level of education, and employment status. Participants older ≥80 y were more likely to rate their sleep duration as being insufficient compared to 40–49-year-olds (p = 0.025); obese individuals were more likely to rate their sleep as being insufficient compared to normal weight individuals (p = 0.001); those with 8 to 11 years of education (p = 0.001) and those with 12 +years of education (p = 0.017) were less likely to rate their sleep as being insufficient compared to those with no formal education; and homemakers were less likely to rate their sleep as being insufficient compared to those not working (p < 0.001).

Ratings of restless sleep were associated with age, sex, education level, employment and marital status. Individuals ≥50 y were more likely to report restless sleep compared to those aged 40–49 y (p < 0.001); females were more likely to report restless sleep than males (p = 0.001); those with ≥ 12 years of education were less likely to report restless sleep compared to those with no formal education (p < 0.001); employed individuals were less likely to report restless sleep compared to those not working (p = 0.011); and separated/divorced (p = 0.002) and widowed (p < 0.001) participants were more likely to report restless sleep compared to married participants.

Self-reported snoring was associated with age, sex, BMI, education level and marital status. Participants ≥ 50 y were more likely to report snoring than 40–49-year-olds (p = 0.044 for 50–59 y, p < 0.001 for 60–69 y, p = 0.004 for 70–79 y, p < 0.001 for ≥ 80 y); overweight (p = 0.009) and obese (p < 0.001) individuals were more likely to report snoring compared to normal weight individuals; males were more likely to report snoring than females (p < 0.001); those with 1–7 y of education were more likely to report snoring compared to those with no formal education (p = 0.005); and separated/divorced (p = 0.031) and widowed (p < 0.001) participants were less likely to report snoring compared to those currently married.

### Sleep and health outcomes

Associations between self-reported sleep parameters and health outcomes are presented in Table 3. Obesity was associated with self-reported bedtime, wake time, insufficient sleep, snoring, gasping and periods of stopping breathing during sleep. Those reporting bedtimes between 21:00–21:30 (p = 0.012) and wake times from 05:00–06:00 (p = 0.019) were more likely to be obese than those going to bed before 21:00 and waking up before 05:00 respectively. In addition, those reporting not getting enough sleep (p = 0.008), snoring (p < 0.001), gasping (p < 0.001) and have breathing pauses (p < 0.001) during the night were also more likely to be obese. Hypertension was associated with self-reported sleep duration, bedtime, wake time, sleep quality, restless sleep, and periods of stopping breathing during the night. In this cohort, those with earlier bedtimes (<21:00, p = 0.028), early waking times (<05:00, p = 0.030), better sleep quality (p = 0.010), less restless sleep (p = 0.011) and those not reporting periods of stopping breathing (p = 0.009) were less likely to have hypertension.

**Table 3:** Logistic regression models for associations between sleep parameters and health conditions.

		Obesity	Hypertension	Dyslipidemia	Diabetes	Anemia	HIV
		OR (95% CI)	OR (95% CI)	OR (95% CI)	OR (95% CI)	OR (95% CI)	OR (95% CI)
(1) Self-reported sleep duration	Quartile 1 (4–7 h) vs 2 (8 h)	0.97 (0.82, 1.17)	1.18 (1.00, 1.39)	0.94 (0.79, 1.12)	1.12 (0.86, 1.47)	1.26 (1.01, 1.58)	1.05 (0.86, 1.29)
	Quartile 3 (9 h) vs 2 (8 h)	1.06 (0.87, 1.30)	1.12 (0.93, 1.35)	0.99 (0.82, 1.20)	1.02 (0.75, 1.38)	1.19 (0.92, 1.52)	1.19 (0.95, 1.49)
	Quartile 4 (10–14 h) vs 2 (8 h)	1.07 (0.86, 1.33)	0.84 (0.69, 1.03)	0.88 (0.72, 1.09)	1.31 (0.96, 1.79)	1.43 (1.10, 1.86)	1.08 (0.84, 1.38)
(2) Bedtime	Quartile 2 (8- <9 pm) vs 1 (<8 pm)	1.22 (0.96, 1.55)	1.15 (0.94, 1.42)	0.94 (0.75, 1.17)	1.01 (0.72, 1.40)	0.96 (0.73, 1.26)	1.35 (1.03, 1.76)
	Quartile 3 (9–9:30 pm) vs 1 (<8 pm)	1.36 (1.07, 1.73)	1.26 (1.02, 1.56)	1.10 (0.88, 1.37)	0.80 (0.57, 1.12)	0.99 (0.75, 1.30)	1.21 (0.93, 1.58)
	Quartile 4 (>9:30 pm) vs 1 (<8 pm)	1.24 (0.96, 1.60)	1.11 (0.89, 1.39)	0.96 (0.76, 1.22)	0.68 (0.47, 0.99)	0.96 (0.71, 1.28)	1.14 (0.86, 1.52)
(3) Wake time	Quartile 2 (5 am) vs 1 ( < 5 am)	1.15 (0.95, 1.40)	0.81 (0.68, 0.98)	1.01 (0.83, 1.22)	1.22 (0.91, 1.64)	0.95 (0.75, 1.21)	0.92 (0.74, 1.15)
	Quartile 3 (>5–6 am) vs 1 (<5 am)	1.27 (1.04, 1.55)	0.98 (0.81, 1.18)	1.07 (0.88, 1.30)	1.05 (0.77, 1.43)	1.00 (0.79, 1.28)	1.14 (0.91, 1.42)
	Quartile 4 (>6 am) vs 1 (<5 am)	1.23 (0.97, 1.57)	0.96 (0.77, 1.19)	1.02 (0.81, 1.27)	1.36 (0.96, 1.93)	1.14 (0.85, 1.51)	0.99 (0.75, 1.29)
(4) Sleep quality	Bad vs good	0.82 (0.61, 1.11)	1.43 (1.08, 1.90)	1.28 (0.98, 1.68)	1.08 (0.72, 1.64)	1.33 (0.96, 1.84)	0.76 (0.53, 1.09)
(5) Latency > 30 min	At least once/week vs. <once/week	0.97 (0.84, 1.12)	1.08 (0.94, 1.23)	1.01 (0.88, 1.16)	1.10 (0.89, 1.36)	1.09 (0.91, 1.30)	0.93 (0.78, 1.09)
(6) Insufficient sleep	Sometimes/rarely/never sufficient vs often/very often sufficient	1.21 (1.05, 1.40)	1.10 (0.96, 1.26)	1.16 (1.01, 1.33)	0.89 (0.72, 1.10)	1.05 (0.88, 1.25)	0.93 (0.79, 1.09)
(7) Restless sleep	Yes vs no	0.95 (0.82, 1.10)	1.19 (1.04, 1.37)	1.06 (0.92, 1.22)	1.29 (1.05, 1.60)	1.04 (0.87, 1.25)	0.86 (0.72, 1.02)
(8) Nocturnal awakenings	At least once/week vs < once/week	0.96 (0.84, 1.10)	1.12 (0.98, 1.27)	0.97 (0.85, 1.10)	1.02 (0.83, 1.25)	0.99 (0.84, 1.18)	1.09 (0.93, 1.27)
(9) Snoring	Yes vs no	2.18 (1.85, 2.57)	1.15 (0.98, 1.35)	0.97 (0.82, 1.14)	1.41 (1.12, 1.78)	0.82 (0.65, 1.02)	0.89 (0.72, 1.09)
(10) Gasping	Yes vs no	2.13 (1.75, 2.59)	1.07 (0.88, 1.30)	0.93 (0.76, 1.13)	1.27 (0.97, 1.67)	0.90 (0.69, 1.18)	1.05 (0.82, 1.34)
(11) Breathing stops	Yes vs no	1.77 (1.37, 2.30)	1.42 (1.09, 1.85)	0.94 (0.72, 1.21)	1.29 (0.91, 1.83)	1.10 (0.79, 1.53)	0.85 (0.60, 1.20)

Each of the six health conditions (dependent variables) was evaluated in a separate model for each off the 11 sleep variables (independent variables), adjusted for BMI category and demographic characteristics (age group, sex, education group, employment status, marital status, wealth index quintile). The obesity outcome was not adjusted for BMI.

OR: odds ratio, CI: confidence interval, BMI: body mass index.

Diabetes was associated with bedtime, restless sleep, and snoring. Participants reporting the latest bedtimes (>21:30) were less likely to have diabetes compared to those with the earliest bedtimes (<20:00, p = 0.047); those reporting restless sleep were more likely to have diabetes than those not reporting restless sleep (p = 0.015); and those reporting snoring were more likely to have diabetes compared to those not reporting snoring (p = 0.003). Dyslipidaemia was only associated with sleep sufficiency such that those reporting insufficient sleep were more likely to have dyslipidemia compared to those who felt they were obtaining sufficient sleep (p = 0.032). Anaemia was associated with self-reported sleep duration such that individuals in the shortest (4–7 h, p = 0.041) and longest (>= 10 h, p = 0.007) sleep quartiles were more likely to have anaemia compared to those in the second sleep quartile (8 h). HIV infection was associated with bedtime only: participants with moderately early bedtimes (20:00 to <21:00) were more likely to be HIV positive compared to those with the earliest bedtimes (<20:00, p = 0.025).

### ART and viral load associations with sleep parameters

While HIV positive individuals not on ART reported more awakenings during the night compared to those on ART (p = 0.024; Table 4), viral load amongst the HIV positive participants was not associated with any of the self-reported sleep parameters measured (Supplementary Table 1).

**Table 4:** Effect of HIV status and current antiretroviral therapy (ART) use on sleep characteristics among the entire cohort.

	Self-reported sleep duration	Bad sleep quality	Insufficient sleep	Restless sleep	Awakenings	Snoring	Gasping	Breathing stops
	Quartile 1 - Quartile 4	Very bad/Bad vs. Very good/Good	Sometimes/Rarely/Never sufficient vs Often/Very often sufficient	Yes vs No	At least once per week vs < Once per week	Yes vs No	Yes vs No	Yes vs No
	OR (95% CI)	OR (95% CI)	OR (95% CI)	OR (95% CI)	OR (95% CI)	OR (95% CI)	OR (95% CI)	OR (95% CI)
HIV+/ART+vs HIV−	1.10 (0.93, 1.29)	0.74 (0.49, 1.14)	0.99 (0.82, 1.20)	0.87 (0.71, 1.06)	1.00 (0.83, 1.21)	0.87 (0.68, 1.11)	1.06 (0.79, 1.41)	0.71 (0.46, 1.10)
HIV+/ART− vs HIV−	0.97 (0.79, 1.20)	0.84 (0.50, 1.42)	0.80 (0.62, 1.03)	0.88 (0.68, 1.13)	1.30 (1.04, 1.64)	0.91 (0.67, 1.23)	1.00 (0.68, 1.44)	1.09 (0.68, 1.74)

Models were estimated using ordered logistic regression with the sleep variables treated as dependent variables in each of the 8 models. Models were adjusted for BMI and demographic characteristics (age group, sex, education group, employment status, marital status, wealth index quintile).

OR: odds ratio, CI: confidence interval.

## Discussion

This cohort of older, rural, black South African adults reported a longer sleep duration (8.2 ± 1.6 h per night) than observed in other populations globally, despite the fact that one third described their sleep duration as being insufficient. A study of self-reported sleep duration and sleep problems in older adults found that participants from South Africa reported sleep durations of 8.6 h (SD ± 2.1), and a greater proportion of rural dwelling participants than urban dwellers reported sleep durations >10 h^25^. A review of data from 168 studies around the world (n = 6052) between 2010 and 2015 found total sleep time (actigraphy- or PSG-derived) to range between 5.8 and 7.8 h per night^26^. Other large-scale epidemiological studies have observed self-reported median sleep durations of 7–8 h^27–30^. Although self-reported sleep duration is typically longer than objectively measured total sleep time, possible explanations for the longer self-reported sleep duration observed in this study may relate to differences in ethnicity (or tribal origin), geographical region, culture, proportion of females, age, employment status, level of education, or a rural living environment.

There may be a strong argument relating to ethnicity, or culture, since three other South African cohorts have also found longer self-reported sleep durations (8.5–10.5 h per night) in black South African adults^19,20,31^. Since a previous study^20^ reported longer sleep durations in black Africans than in the other ethnic groups living in South Africa, it is tempting to surmise that a longer self-reported sleep duration is particular to ethnicity or culture in this setting. Interestingly, data from the 2005–2012 National Health and Nutrition Examination Survey (NHANES) in the USA indicated that non-Hispanic black Americans reported shorter sleep durations than other ethnic groups^32^. Furthermore, a meta-analysis comparing the sleep of African-Americans to European-Americans using polysomnography, actigraphy, diaries and questionnaires found that African-Americans had shorter total sleep time (objectively and subjectively measured) and poorer quality sleep, as measured by worse sleep efficiency, longer sleep onset latency and less slow wave sleep^33^. Therefore, it is important to try to disentangle the factors underlying these variations between ethnic groups in different countries with disparate socio-economic histories which appear to reoccur in spite of variability in general lifestyle and culture.

Longer self-reported sleep duration has been shown to be more prevalent in adults younger than 25 years or older than 60 years in the United States^34^. The longer self-reported sleep duration observed in the present study may be, at least in part, due to the older age of the cohort. Counter to this argument, however, is that those between 50 and 70 years old in this study reported shorter sleep durations than the 40-year-olds, and the sleep durations reported in previous South African studies, 8.75 h^19^ and 10.35 h^31^, were both longer than this study, despite the younger ages of their participants (39.9 years and 33.4 years respectively). There is evidence to suggest that males typically have longer, better quality sleep than females (reviewed in^35^). Since our sample was mildly biased towards females (54%), whose average sleep duration was 8.15 h, sex seems unlikely to explain the longer self-reported sleep duration observed in this cohort.

Another point to consider relates to employment. A study of adults in the USA found that being employed had a strong effect on reducing self-reported sleep duration^34^. Since only 16% of the individuals in the present study were employed, the longer sleep durations are not unexpected, and indeed employed individuals from this cohort did report shorter sleep durations. At odds with our observation is that the Soweto cohort, in whom the employment rate was nearly double (30%), reported longer sleep durations^19^. Of interest, too, is that despite the lower proportion of females in paid employment in this study, they reported earlier rise times and shorter sleep durations than the males. This is the opposite of what was observed in Soweto, Finland, Norway, the UK and Canada^19,27,36,37^.

One might also argue that the longer sleep durations reported in this study relate to a rural living environment. Norwegian^36^ and South African studies^25^ have observed that individuals living in rural areas reported longer sleep durations than those in urban areas. However, the fact that an urban, albeit younger, population in Soweto, South Africa reported a longer sleep duration (8.8 ± 1.7 h)^19^, counters this argument. Generally, self-reported sleep durations from native Africans living in South Africa — those reported in the Soweto cohort^19^, the black African participants from the SAGE cohort^25^ and the black South Africans from the Modelling the Epidemiological Transition Study (METS) cohort^31^ — are all similar to those reported in the present study. We note that the sleep durations reported in this study are also similar to the objectively measured sleep periods (i.e. time-in-bed) in rural (9.23 ± 0.13 h) and semi-rural (8.78 ± 0.18 h) African communities in Mozambique^18^ but longer than those measured in southern African hunter-gathers (6.9–8.5 h) (Yetish, 2015, #3241}. We interpret these comparisons, with caution, however, owing to the different data collection methods used.

Thus, these findings raise interesting questions. Do South Africans, and in particular those of black African ancestry, have different sleep habits or even different sleep needs compared to other populations? Are these self-reported findings truly indicative of longer sleep, or could self-reported sleep in this cohort be subject to interpretation bias? Could this longer sleep period indicate sleep quality issues? What does the sleep period and NCD risk profile association look like in this group? To truly address these questions, future studies making use of objective sleep measures are required. The regression analyses from the present study do, however, provide some insight to the latter questions.

Data from the WHO indicate that the two strongest contributing risk factors to NCD-related deaths in South Africa are elevated blood pressure (33.7%) and obesity (31.3%)^38^. This sample of rural, older, longer sleeping adults was on average overweight, and had a high prevalence of obesity (30%) and hypertension (58%). Similar to the SA-NHANES report^1^, the incidence of obesity and hypertension was higher in the females in this study than in the males. The females also had higher incidences of depression and anaemia than the males. This higher burden of disease in the females was coincident with less formal education, larger households and lower employment. Since they also spent fewer hours in bed, had earlier wake times and reported more restless sleep and nocturnal awakenings than the males, one might speculate that their shorter, poorer quality sleep places them at higher risk for cardiometabolic diseases.

Prospective cohort studies indicate that both long- and short-total sleep times are associated with higher mortality rates and risk for cardiometabolic diseases^10–13,39^. In a recent large-scale study in the USA, 7 h of sleep was associated with the lowest risk for cardiometabolic disease in all ethnic groups except non-Hispanic blacks, in whom 8 h of sleep was associated with the lowest risk score for cardiometabolic disease^32^. As in previous studies^40,41^, the long sleepers (>10 h) in the present study had a lower incidence of hypertension than the shorter sleepers (<7 h). Sleep timing and ratings of sufficiency also appear to be important contributors to obesity in this cohort, since we observed an association between later bedtimes, ratings of insufficient sleep and obesity. A previous study in a cohort of black South Africans living in Soweto reported that a longer total sleep time and naps appeared to be protective against a high BMI in older females and high blood pressure, BMI and abdominal obesity in males^19^. Collectively, these data suggest that optimal sleep need may well differ by ethnicity in the context of minimising risk for NCDs. It is also worth noting that although we did not assess sleep apnoea, there are indications that this cohort may suffer from the condition, which may be contributing to poorer health since those reporting snoring, gasping or having breathing pauses during the night were more likely to be obese.

The relationship between HIV infection, and its treatment, is complex. Sleep disturbances are a known effect of HIV infection^3^. This occurs already at an early stage of the infection, and has been suggested to be associated with chronic activation of the immune system^4,42^. HIV infection in itself has neurotoxic effects, quite apart from the effects of any opportunistic pathogens (reviewed in^43^). It is also well known that patients taking efavirenz, one of the common ART for HIV, experience insomnia and other sleep disturbances such as longer sleep onset latency and shorter sleep duration, in particular in the earlier stages of treatment^5,6^. Electroencephalogram monitoring has shown longer sleep latency and shorter duration of deep sleep in patients taking efavirenz^5^, with higher plasma concentrations of efavirenz associated with insomnia^5,7^. Although it has been reported that some patients stop taking efavirenz as they find the sleep disturbances too debilitating^7,44^, long-term studies find that for most patients these and other neurological and psychiatric side-effects are transient and do not last beyond the first four weeks of treatment^45,46^. Further, the immunoprotective effects of ART have a paradoxical effect when successful treatment commences at an advanced stage of the infection. It has been shown that a high increase in CD4 counts, although a sign of restored immune function, also associates with lower sleep quality (as well as higher incidence of pain and depression)^42^. Our finding that participants with untreated HIV infection and a high viral load reported more nocturnal awakenings than HIV negative participants (p = 0.024) is consistent with previous reports of an effect of the infection on sleep quality. Our finding of less self-reported sleep disturbance in treated than in untreated HIV positive participants are not suggestive of a negative net effect on sleep of ART treatment, either via the effects of the ART drugs or via an asssociated CD4 rebound, in this older population. Although based on self-reported data on sleep quality and quantity, these data do not support any suggestion of replacing efavirenz as first-line treatment specifically because of its effects on sleep, at least in older patients. As with other aspects of these findings, objective measures of sleep parameters, and cellular markers of immune function status would be required in order to determine whether there are effects that are not captured by questionnaires and categorical data on treatment and viral load. Additionally, the present study did not examine other neuropsychiatric symptoms, or younger patients.

In instances where a longer than expected total sleep time is reported, it is reasonable to investigate quality since long sleep may be an indicator for disrupted or inefficient sleep, or an underlying medical condition^47^. In the present study, although the vast majority of participants considered their sleep to be of good quality. There were indications that quality was suboptimal, since one-third took longer than 30 minutes to fall asleep at night and one-third experienced restless sleep. Those with restless sleep were more likely to have hypertension and diabetes, suggesting that sleep quality warrants further investigation in this population.

A limitation of this study is that data collection took place throughout the year across all seasons, since participants were part of the parent HAALSI study. Thus, seasonal variation in photoperiod, with daylight hours in Agincourt ranging between 10h37min and 13h39min, may have influenced self-reported total sleep time. Although this is a rural community, most (but not all) participants have access to electricity and thus light at night. In general, this population rose at, or before, sunrise and remained awake for a few hours after sunset, similar to what has been observed in other rural African populations^16,18^. We were unable to obtain data on exposure to electricity at night, which would have been a helpful covariate in interpretation of these results. Further studies distinguishing between participants with and without access to electricity would be of interest, and would need to be performed before electrification of the region has been completed.

In conclusion, this cohort has the potential to address a number of important questions of current high interest. What is not known is whether the longer total sleep time, and higher prevalence of long sleep (9 h or more) seen in this and other South African studies (i) reflects a greater sleep need, (ii) is a consequence of higher levels of unemployment, (iii) is protective against NCDs, and/or (iv) is underpinned by society-specific beliefs or value placed on sleep in South Africans of African origin. The data presented here provide a basis for further objective studies of the relationship between sleep quality and quantity to other risk factors in South African populations, a complex and underexplored area of research^48^. On a more general note, it also provides valuable data about sleep habits in Africa, with the potential for future analysis of how it is affected by urbanisation and industrialisation. Finally, it offers a unique opportunity for a population-based comparison of the effects of treated and untreated HIV infection, which is increasingly unavailable elsewhere.

## Methods

### Setting

The HAALSI study^23^ was implemented in the Agincourt sub-district in Mpumalanga Province, South Africa, where the MRC/Wits Rural Public Health and Health Transitions Research Unit has been running the Agincourt Health and Demographic Surveillance System (Agincourt HDSS) site since 1992^21^. In 2015, the study area of around 450 km^2^ included 31 villages with a population of approximately 116000 people. The primary health care system consists of six clinics, one public health center and one private-public community health center. The three hospitals that cover the district are 45–60 km from the study site. The social situation of this community and its services have improved in the past 20 years, but there are still gaps in areas such as electricity, water availability and paved roads among other important services^21^. Unemployment is highly prevalent, putting stress on families and leading to high rates of work migration and reliance on remittances as an important source of income^49^. Details on how census rounds were completed, and available information at the individual and household levels have been described elsewhere^21^.

### Participants

The HAALSI participants were randomly selected from the MRC/Wits Agincourt Unit’s annual census of 2013. We defined the inclusion criteria as all males and females 40 years and older as of July 1, 2014, and permanently living in the study site during the 12 months prior to the census round of 2013. This sample included individuals randomly selected to participate in previous studies on ageing and adult health that were run in Agincourt HDSS in 2006^50^ and 2010^51^ to allow potential longitudinal analysis in this sub-sample of HAALSI participants. A sample of 6281 individuals who met the inclusion criteria was selected. Of these, 391 individuals were excluded (deceased or had migrated). The response rate of the 5890 eligible individuals was 85.9%, resulting in a final sample size of 5059^23^. All participants belonged to the Shangaan ethnic group.

### Study design

This is a cross-sectional, observational study in which each participant was interviewed at their home by a trained fieldworker. Interviews were performed using a computer-assisted personal interview (CAPI) on a laptop. The individual interview included an extensive set of questions on socio-demographic variables, self-reported health, and wellbeing, as well as anthropometric, blood pressure, and point of care measurements for glucose, lipids, haemoglobin, and HIV.

### Outcome measures

This investigation conformed with the tenets of the Declaration of Helsinki and was approved by the University of the Witwatersrand Human Research Ethics Committee (#M141159), the Harvard T.H. Chan School of Public Health Office of Human Research Administration (#13–1608), and the Mpumalanga Provincial Research and Ethics Committee. Written informed consent was obtained from all participants. For illiterate participants a witness not linked to the study was present during the consenting process. The socio-demographic questions included information on age (presented in 10-year age bins), sex, years of completed education (no formal education, 1–7 y, 8–11 y, 12 or more years), marital status (never married, currently married, separated/divorced, widowed), employment status (not working, employed, homemaker), and socio-economic status (SES) as household wealth index quintiles^52^. SES was measured using the absolute values of a household asset score, based on five asset categories – ‘modern assets’, ‘power supply’, ‘water and sanitation’, ‘quality of housing’, and ‘livestock assets’ – with increasing values corresponding to greater SES. This score was then divided in quintiles by household.

Anthropometric measurements included height (m), weight (kg), waist and hip circumferences (cm). BMI was calculated and participants classified as obese for BMI ≥ 30 kg/m^2^. Resting blood pressure (BP) was measured (Omron M6W® automated cuff) and participants classified as hypertensive for diastolic BP ≥ 90 mmHg or systolic BP ≥ 140 mmHg or participants reported current use of medication to treat hypertension^53^. Blood was collected using the finger prick technique for dried blood spots (DBS) and point of care measurements were taken for glucose (CareSense, De Puy Synthes, Rainham, MA), lipids (Cardiochek, PTS Diagnostics, Indianapolis, IN), and haemoglobin (HemoCue, Ängelholm, Sweden). Participants were classified as diabetic if their fasting glucose concentration was >7 mmol/L, or non-fasting concentration was ≥11.1 mmol/L or they reported using medication to control diabetes (American Diabetes Association. 2013). Participants were classified as having dyslipidaemia if they had elevated total cholesterol (≥6.21 mmol/L), or low HDL cholesterol (<1.19 mmol/L), or elevated LDL cholesterol (>4.1 mmol/L), or elevated triglycerides (>2.25 mmol/L) levels, or reported current use of cholesterolaemia treatment at time of interview. Anaemia was diagnosed if haemoglobin concentration was <11 g/dl) 1 49.

HIV status was determined by testing DBS for HIV antibodies with enzyme-linked immunosorbent assays [ELISA; Vironostika Uniform 11 (BioMérieux, Marcy-l'Étoile, France)] and confirmed using the Elecsys® HIV combi PT assay (Roche Diagnostics, Basel, Switzerland) and the ADVIA Centaur® HIV Ag/Ab Combo assay (Siemens Healthcare Diagnostics, Norwood, MA). Among those who tested HIV positive, assays were performed for viral load and to detect presence of ART. The Division of Clinical Pharmacology Laboratory of the University of Cape Town performed the ART assays, which detected metabolites of emtricitabine (FTC) or lamivudine (3TC) at a concentration as low as 0.02 µg/ml^50^. Either FTC or 3TC or both are included in all standard first- and second-line ART regimens in South Africa, so presence of metabolites of either drug was used to define those currently on ART.

A subset of the major individual components of the Pittsburgh Sleep Quality index (PSQI)^24^ were analysed in this study, in addition to a number of additional questions. All questions were translated from English to Shangaan and then back translated to English for consistency. The questionnaire in English is listed in Table 5 and the Shangaan version in Annex A. For the purposes of this study the question “Over the past 4 weeks, how many hours do you think you actually slept each day?” was used to determine self-reported sleep duration.

**Table 5:** Questionnaire items used in this study, with response options in parentheses.

• Over the past 4 weeks, what time did you usually turn the lights off to go to sleep? (Hours, minutes)
• Over the past 4 weeks, what time did you usually get out of bed? (Hours, minutes)
• Over the past 4 weeks, how many hours do you think you actually slept each day? (Hours)
• During the past 4 weeks, how often did you wake up in the middle of the night or early morning? (“Never”/”Less than once per week”/”Once or twice a week”/”Three of more times a week”)
• During the past month, have you snored, or ever been told that you were snoring? (“Yes”/”No”)
• During the past month, have you snored loudly, or ever been told that you were snoring loudly? (“Yes”/”No”)
• During the last month, have you had, or ever been told that your breathing stops or you struggle for breath? (“Yes”/”No”)
• During the last month, have you had, or ever been told that you were snorting or gasping? (“Yes”/”No”)
• On a scale of 0 to 10, where 0 is “Does not interfere” and 10 is “Completely interferes”, select the one number that describes how, during the past 24 hours, pain has interfered with your sleep.
• Over the past 4 weeks, how would you rate your sleep quality overall? (“Very good”/”Fairly good”/”Fairly bad”/”Very bad”)
• During the past 4 weeks, how often could you not get to sleep within 30 minutes? (“Never”/”Less than once per week”/”Once or twice a week”/”Three of more times a week”)
• In your life, have you ever had any experience that was so frightening, horrible, or upsetting that, in the past 30 days you had more trouble than usual falling asleep or staying asleep?
• Much of the time in the past week, your sleep was restless. (“Yes”/”No”)
• How often during the past 4 weeks did you get enough sleep to feel rested upon waking up? (“Never”/”Rarely”/”Sometimes”/”Often”/”Very often”)

### Data and statistical analyses

Data collected in the field were checked regularly for quality and completeness following HAALSI standard operating procedures. Descriptive data are presented as mean ± standard deviation, median (interquartile range) or count (%). Sex comparisons were carried out using an independent t-test, a Mann-Whitney U test or a Fisher’s Exact test. The association of demographic variables and sleep parameters were measured using logistic regression. Models for continuous variables were estimated using ordered logistic regression, with sleep duration, bedtime and wake time quartiles as the dependent variables. Quartiles of sleep duration were 4–7 h (quartile 1, n = 1,542), 8 h (quartile 2, n = 1,271), 9 h (quartile 3, n = 1,007), and 10–14 h (quartile 4, n = 853). The quartiles for bed time were earlier than 20:00 (quartile 1, n = 667), 20:00 to <21:00 (quartile 2, n = 1,602), 21:00–21:30 (quartile 3, n = 1,622), and later than 21:30 (quartile 4, n = 1,109). The quartiles for wake time were earlier than 05:00 (quartile 1, n = 887), 05:00 (quartile 2, n = 1,626), >05:00 to 06:00 (quartile 3, n = 1,537), and later than 06:00 (quartile 4, n = 893). We then modelled the association of sleep parameters and health conditions (hypertension, dyslipidaemia, diabetes, anaemia and HIV) also using logistic regression. In these analyses, each sleep parameter was evaluated in a separate model, adjusted for BMI and demographic characteristics (age group, sex, education group, employment status, marital status, wealth index quintile). For those participants who were HIV positive, we studied the association of being on ART and viral load on the main sleep variables (duration, quality, quantity, restless, awaking at night, snoring, gasping, nocturnal apnea). These models were adjusted for BMI and demographic characteristics (age group, sex, education group, employment status, marital status, wealth index quintile). Data were analysed using SAS software v9.3, and significance was accepted for p < 0.05.

## Electronic supplementary material

Supplement Table 1 and Table 5 Supplement

## Data Availability

The baseline data used in this study is publicly available at the Harvard Center for Population and Development Studies (HCPDS) program website (www.haalsi.org). The data is also accessible through the Interuniversity Consortium for Political and Social Research (ICPSR) at the University of Michigan (www.icpsr.umich.edu), and the INDEPTH Data Repository (http://www.indepth-ishare.org/index.php/catalog/113). Further details can be obtained by emailing the corresponding author of this paper.
