# Supplementary material for: Associations between sleep parameters, non-communicable diseases, HIV status and medications in older, rural South Africans
Source: Sci Rep. 2018 Nov 23;8:17321. doi: 10.1038/s41598-018-35584-0 (PMC6251877; doi:10.1038/s41598-018-35584-0)
Supplement: Supplementary file 1 — Supplement Table 1 and Table 5 Supplement [file 41598_2018_35584_MOESM1_ESM.docx]

**Associations between sleep parameters, non-communicable diseases, HIV status and medications in older, rural South Africans**

F. Xavier Gómez-Olivé^1,2,3^*, Julia K Rohr^2^, Laura C Roden^4^, Dale E Rae^5^, Malcolm von Schantz^6^

^1^ MRC/Wits Rural Public Health and Health Transitions Research Unit (Agincourt), School of Public Health, Faculty of Health Sciences, University of the Witwatersrand, Johannesburg, South Africa

^2^ Harvard Center for Population and Development Studies, Harvard T.H. Chan School of Public Health, Harvard University, Cambridge, Massachusetts, USA

^3^ INDEPTH Network, Accra, Ghana

^4^ Department of Molecular and Cell Biology, Faculty of Science, University of Cape Town, Cape Town, South Africa, Laura.Roden@uct.ac.za

^5^ Division of Exercise Science and Sports Medicine, Department of Human Biology, Faculty of Health Sciences, University of Cape Town, Cape Town, South Africa, Dale.Rae@uct.ac.za

^6^ Faculty of Health and Medical Sciences, University of Surrey, Guildford, Surrey, UK, m.von.schantz@surrey.ac.uk

***Corresponding author:**

F. Xavier Gómez-Olivé. [f.gomez-olivecasas@wits.ac.za](mailto:f.gomez-olivecasas@wits.ac.za). School of Public Health Building,

University of the Witwatersrand (Education Campus), 27 St Andrews Road Parktown, 2193 South Africa

**Supplement Table 1**. Effect of antiretroviral therapy (ART) and viral load on sleep characteristics among HIV+ people (N=1035). Models were estimated using ordered logistic regression with the sleep variables treated as dependent variables in each of the 8 models. Models included ART / viral load category, and were adjusted for BMI and demographic characteristics (age group, sex, education group, employment status, marital status, wealth index quintile).

|  |  | **Self-reported sleep duration** | **Bad sleep quality** | **Insufficient sleep** | **Restless sleep** | **Awakenings** | **Snoring** | **Gasping** | **Breathing stops** |
| --- | --- | --- | --- | --- | --- | --- | --- | --- | --- |
|  |  | Quartile 1 - Quartile 4 | Very bad/ Bad vs Very good/ Good | Sometimes/ Rarely/ Never sufficient vs Often/ Very often sufficient | Yes vs No | At least once per week vs < Once per week | Yes vs No | Yes vs No | Yes vs No |
| **Viral load /ART status** | N (%) | OR (95% CI) | OR (95% CI) | OR (95% CI) | OR (95% CI) | OR (95% CI) | OR (95% CI) | OR (95% CI) | OR (95% CI) |
| ≤1000 copies/mL / ART+ | 595 (57%) | Ref |  |  |  |  |  |  |  |
| >1000 copies/mL / ART+ | 68 (7%) | 1.27 (0.78, 2.07) | 1.38 (0.45, 4.29) | 0.82 (0.45, 1.48) | 0.88 (0.47, 1.63) | 0.89 (0.51, 1.55) | 0.82 (0.37, 1.83) | 1.00 (0.40, 2.50) | 0.37 (0.05, 2.84) |
| ≤1000 copies/mL / ART- | 131 (13%) | 1.00 (0.69, 1.44) | 0.78 (0.26, 2.37) | 0.76 (0.49, 1.17) | 1.01 (0.65, 1.57) | 1.433 (0.96, 2.138) | 1.02 (0.59, 1.74) | 0.89 (0.47, 1.71) | 1.34 (0.57, 3.15) |
| >1000 copies/mL / ART- | 241 (23%) | 0.87 (0.65, 1.17) | 1.42 (0.70, 2.91) | 0.79 (0.56, 1.11) | 0.94 (0.66, 1.35) | 1.26 (0.92, 1.73) | 1.03 (0.66, 1.60) | 0.98 (0.57, 1.66) | 1.49 (0.74, 2.99) |

Note: 1048 individuals tested positive for HIV through dried blood spot (DBS) screening. The model excludes 13 people who were missing data on ART status, for a final N of 1035.

**Table 5 Supplement.** Questionnaire items used in this study, with response options in parentheses and translated to Shangaan, the local language.

- Over the past 4 weeks, what time did you usually turn the lights off to go to sleep? (Hours, minutes)
  - Eka mavhiki ya mune lama nga hundza, xana a mi tala ku tima hi nkarhi muni timboni loko mi etlela? Ndzi byeleni awara na timinete ta siku. Xik: 20:30 pm.
- Over the past 4 weeks, what time did you usually get out of bed? (Hours, minutes)
  - Eka mune wa mavhiki la ma nga hundza, xana a wu tala ku pfuka hi nkarhi muni?
- Over the past 4 weeks, how many hours do you think you actually slept each day? (Hours)
  - Eka mavhiki ya mune la ma nga hundza, xana mi ehleketa leswaku mi etlele tiawara ti ngaki eka siku rinwana na rinwana
- During the past 4 weeks, how often did you wake up in the middle of the night or early morning? (“Never”/”Less than once per week”/”Once or twice a week”/”Three of more times a week”)
  - Eka mavhiki ya mune lama nga hundza, xana a mi tala ku pfuka exikarhi ka vusiku kumbe mi pfuka hi mahlamba-ndlopfu? 1 A swi se tshama (Never) 2 Ehansi ka kanwe evhikini (Less than once a week) 3 Kanwe kumbe ka mbirhi evhikini (Once or twice a week) 4 Ka nharhu kumbe mi karhi yo tala evhikini (Three or more times a week)
- During the past month, have you snored, or ever been told that you were snoring? (“Yes”/”No”)
  - Eka nhweti leyi nga hundza, xana mi bile mantonoro kumbe mi byeriwa ku a mi ba mantonoro? 1 (YES) Ina 2 (NO) Ee
- During the past month, have you snored loudly, or ever been told that you were snoring loudly? (“Yes”/”No”)
  - Eka nhweti leyi nga hundza, xana mi bile huwa hi mantonoro, kumbe mi byeriwile leswaku a mi ba huwa hi mantonoro? 1 (YES) Ina 2 (NO) Ee
- During the last month, have you had, or ever been told that your breathing stops or you struggle for breath? (“Yes”/”No”)
  - Eka nhweti leyi nga hundza, xana mi nga va mi byeriwile leswaku a mi tikeriwa hi ku hefemula? 1 (YES) Ina 2 (NO) Ee
- During the last month, have you had, or ever been told that you were snorting or gasping? (“Yes”/”No”)
  - Eka nhweti leyi nga hundza, xana mi nga va mi byeriwile leswaku a mi ba mantonoro kumbe mi pfule nomo? 1 (YES) Ina 2 (NO) Ee
- On a scale of 0 to 10, where 0 is “Does not interfere” and 10 is “Completely interferes”, select the one number that describes how, during the past 24 hours, pain has interfered with your sleep.
  - Eka mpimo wa 0 ku fika ka 10, laha 0 swi vulaka kuri a swi kavanyeti naswona 10 swi vulaka kuri swi kavanyeta swinene, hlawula nomboro leyi hlamuselaka kuri swivavi swa wena swi nga va swi kavanyete njhani eka vurhongo bya wena eka makume mbirhi mune wa tiawara leti nga hundza.
- Over the past 4 weeks, how would you rate your sleep quality overall? (“Very good”/”Fairly good”/”Fairly bad”/”Very bad”)
  - Eka mune wa mavhiki la ma nga hundza, xana a mi etlela njhani? 1 Kahle ngopfu 2 Kahle ka tsongo 3 A swi bihe ka tsongo 4 A swi bihe ngopfu
- During the past 4 weeks, how often could you not get to sleep within 30 minutes? (“Never”/”Less than once per week”/”Once or twice a week”/”Three of more times a week”)
  - Eka mune wa mavhiki la ma nga hundza, xana I ka ngaki laha a mi nga etleli ku nga se hundza 30 wa timinete? 1 A swi se tshama (Never) 2 Ehansi ka kanwe evhikini (Less than once a week) 3 Kanwe kumbe ka mbirhi evhikini (Once or twice a week) 4 Ka nharhu kumbe mi karhi yo tala evhikini (Three or more times a week)
- In your life, have you ever had any experience that was so frightening, horrible, or upsetting that, in the past 30 days you had more trouble than usual falling asleep or staying asleep?
  - Evutonwini bya wena, xana u nga va u ti kume u chuhile/chava kumbe mi kwatile eka masiku ya makume nharhulama nga hundza, laha u nga ti kuma u ri na xiphiqo xa vurhongo, kumbe nkelunkelu? 1 (YES) Ina 2 (NO) Ee
- Much of the time in the past week, your sleep was restless. (“Yes”/”No”)
  - Nkarhi wotala vhiki leri nga hundza, a mi xanisa hi nkelunkelu. 1 (YES) Ina 2 (NO) Ee
- How often during the past 4 weeks did you get enough sleep to feel rested upon waking up? (“Never”/”Rarely”/”Sometimes”/”Often”/”Very often”)
  - Eka mune wa mavhiki la ma nga hundza, xana i ka ngaki laha u nga ti twa u etlele ku ringanela ku fikela laha loko u pfuka u titwa u wisile? 1 A swi se tshama (Never) 2 A swi talanga (Rarely) 3 Mi karhi yinwana (Sometimes) 4 M karhi yo tala (Often) 5 mi karhi yo tala ngopfu (Very often)
